# Does the Implant Surgical Technique Affect the Primary and/or Secondary Stability of Dental Implants? A Systematic Review

**DOI:** 10.1155/2014/204838

**Published:** 2014-07-07

**Authors:** Rola Muhammed Shadid, Nasrin Rushdi Sadaqah, Sahar Abdo Othman

**Affiliations:** ^1^Department of Prosthodontics, Faculty of Dentistry, Arab American University, Jenin, Palestine; ^2^Department of Oral Surgery, Faculty of Dentistry, Al-Sharjah University, Al-Sharjah, UAE

## Abstract

*Background*. A number of surgical techniques for implant site preparation have been advocated to enhance the implant of primary and secondary stability. However, there is insufficient scientific evidence to support the association between the surgical technique and implant stability. *Purpose*. This review aimed to investigate the influence of different surgical techniques including the undersized drilling, the osteotome, the piezosurgery, the flapless procedure, and the bone stimulation by low-level laser therapy on the primary and/or secondary stability of dental implants. *Materials and methods*. A search of PubMed, Cochrane Library, and grey literature was performed. The inclusion criteria comprised observational clinical studies and randomized controlled trials (RCTs) conducted in patients who received dental implants for rehabilitation, studies that evaluated the association between the surgical technique and the implant primary and/or secondary stability. The articles selected were carefully read and classified as low, moderate, and high methodological quality and data of interest were tabulated. *Results*. Eight clinical studies were included then they were classified as moderate or high methodological quality and control of bias. *Conclusions*. There is a weak evidence suggesting that any of previously mentioned surgical techniques could influence the primary and/or secondary implant stability.

## 1. Introduction

Use of dental implants has become a widespread and predictable treatment modality for the restoration of missing teeth and various edentulous cases [1]. As progress in material and implant design continues dramatically over time, implant patients have been demanding treatment protocols that take less time and require fewer surgeries [2]. Consequently, immediate loading of dental implants has gained popularity and becomes more and more required. A fundamental prerequisite for immediate loading is substantial primary implant stability at the time of insertion [3]. Primary stability is defined as the absence of mobility in the bone bed after the implant has been placed [4]. It depends on mechanical engagement of an implant with the fresh bone socket, but this stability declines with time during the early stages of healing, as remodeling of the surrounding bone takes place [5]. While secondary stability is the progressive increase in stability related to biologic events at the bone-implant interface such as new bone formation and remodeling [6], it is absent at the time of implant placement and increases with time.

In addition to considering the primary implant stability a critical factor when immediate loading is planned [3], it is one of the prerequisites for achievement and maintenance of osseointegration. Besides the quantity and quality of the bone [4, 7], morphology of the implant [4], implant surface roughness, and topography [8, 9], the surgical technique adopted also influences the primary stability [4, 10]. Likewise, secondary stability is mainly influenced by implant characteristics and surgical technique [11].

One of the surgical techniques suggested to enhance the primary stability of implant in bone of low density is the undersized drilling technique, which has been introduced to locally optimize the bone density by using a final drill diameter considerably smaller compared with the implant diameter [12]. In this way, an osteocompressive fit between the implant surface and bone bed is achieved. However, a drawback of all drilling techniques is that bone tissue is sacrificed during the drilling process. This shortcoming is exacerbated in situations where limited bone or bone of lesser density is available [13]. In view of this, the osteotome technique has been introduced [14]. This technique consists of first preparing a small-sized pilot hole, then compressing the bone tissue laterally and apically with a spreader or implant-shaped instrument. The goal of this technique is to replace the implant with a high degree of stability without removing additional bone, which is theoretically believed to improve final bone healing [15–17].

In addition to conventional surgical preparation techniques, the piezoelectric bone surgery [18, 19] offers an alternative technique to implant placement that professes to address some of the shortcomings of the conventional system utilizing an ultrasonic surgical system. The piezoelectric surgery unit claims to be superior to conventional methods in numerous ways: improved precision, selective cutting action, minimal damage to soft tissues such as nerves or blood vessels, reduced bleeding resulting in improved visibility within the surgical field, and the absence of overheating [19, 20]. Currently, the effect of ultrasounds is being widely investigated in various fields of medicine: in orthopedics, they are used to accelerate healing of bone fractures and ligament damage by promoting cell proliferation and bone matrix synthesis [21–23]. Also, multidisciplinary clinical reports on the application of ultrasounds in bone surgery obtained promising results in terms of precision and safety [24, 25].

The use of low-level lasers has also been suggested as another way of accelerating and improving the bone tissue healing process [26]. Laser light irradiation has been applied in the medical field and has biostimulatory effects on wound healing, collagen synthesis, and fibroblast proliferation [27–29]. In addition, it has been demonstrated that bone irradiated mostly with infrared wavelengths shows increased osteoblastic proliferation, collagen deposition, and bone formation when compared to nonirradiated bone [30, 31]. However, little reliable data exist concerning the laser effect on the osseointegration process of implants. Therefore, this review aimed to find if there is scientific-based evidence to support laser influence on stability of dental implants.

Nowadays, various computer-assisted systems comprising a three-dimensional virtual planning by means of a computer tomography (CT) or digital volume tomogram suggest a flapless procedure [32]. For the accurate and predictable placement of the implants, a surgical template is fabricated based on the virtual planning and consequently a prosthetically driven and template-guided implant placement can be carried out [33]. According to Oh et al. [34], a flapless implant surgery provides esthetic soft tissue results in single-tooth implants either immediately or delayed loaded. It has been documented that the use of stereolithographic appliances in accordance with flapless surgery assists in the immediate loading of implants [35]. Also, immediate loading with flapless surgery technique has been shown to reduce the treatment period and enhance implant stability compared to the conventional flap surgery protocol [36, 37].

Different clinical methods for monitoring implant stability at various stages have been proposed, such as Periotest (Siemens AG, Bensheim, Germany), Dental Fine Tester (Kyocera, Kyoto, Japan), Osstell Mentor (Osstell AB, Stampgatan, Göteborg, Sweden), and the cutting-torque or insertion torque (IT) measurement [38]. The Periotest is composed of a metallic tapping rod in a handpiece, which is electromagnetically driven and electronically controlled. Signals produced by tapping are converted to unique values called “Periotest values (PTV)” [4]. These values range from −8 to +50. The lower the PTV, the higher the implant stability [7]. However, Periotest and Dental Fine Tester have been the subject of criticism as a result of their poor sensitivity and because their measurements are significantly influenced by variables such as the vertical measuring point on the implant abutment, the handpiece angulations, and the horizontal distance of the handpiece from the implant [39, 40]. The Osstell resonance frequency analysis (RFA) system involves the placement of a Smart Peg into the implant, which is screwed into the implant itself and the use of a transducer, which is held close to and perpendicular to the Smart Peg without actually making contact. Custom Smart Pegs are available for all major implant systems [41]. Osstell gives the implant stability quotient (ISQ) through resonance frequency analysis on a scale from 1 to 100. The higher the ISQ number, the higher the stability [7]. Recent studies have shown the ISQ to be an accurate, noninvasive means of determining implant stability, and it is becoming a widely used instrument [42–44] to monitor the changes in stiffness and stability at the implant-tissue interface and to discriminate between successful implants and clinical failures [44, 45]. Regarding the cutting-torque or insertion torque measurement which was originally developed by Johansson and Strid [38] and later improved by Friberg et al. [46], its main purpose was to quantify the initial torque (in Ncm units) required to seat the implant into the socket during surgery by means of a torque application device (OsseoCaret) and thereby predict bone support and density [5]. Although this method is nonsubjective, noninvasive, and extensively used in clinical practice during implant placement to assess primary stability [47], it allows only a single measurement at implant insertion and cannot be used for evaluating secondary stability [7].

Considering that the surgical technique might influence the primary and secondary stability of dental implants, the aim of this systematic review was to investigate the influence of different surgical techniques including the undersized drilling, the osteotome, the piezosurgery, the flapless procedure, and the bone stimulation by low-level laser therapy on the primary and/or secondary stability of dental implants.

The present systematic review was focused on this question: is there scientific evidence to support the influence of these surgical techniques on the primary and/or secondary stability of dental implants?

## 2. Methods

### 2.1. Criteria for Considering Studies for this Review

The inclusion criteria comprised observational clinical studies and randomized controlled trials (RCTs) conducted in patients who received dental implants for rehabilitation, studies that evaluated the association between the surgical technique (prognostic factor) and implant primary and/or secondary stability (outcome). Surgical techniques evaluated were the underdrilling technique, osteotome technique, piezosurgery, flapless technique, and the low-level laser therapy. Dental implant stability was evaluated by ISQ value (Osstell, Integration Diagnostics, Gothenburg, Sweden), PTV value (Periotest, Medizintechnik Gulden, Modautal, Germany), or IT measurement. Secondary implant stability should be recorded at least three months after implant placement. Studies that reported surgical technique and implant stability but did not verify their association were excluded from this systematic review.

### 2.2. Search Method for Identification of Studies

For the identification of the clinical studies to be considered in this review, combinations of the following keywords were used: “dental implants,” “implant stability,” “primary stability,” “secondary stability,” “implant stability quotient,” “ISQ,” “resonance frequency analysis,” “RFA,” “Osstell,” “Periotest value,” “PTV,” “Periotest,” “osteotome technique,” “undersized drilling,” “piezosurgery,” “surgical technique,” “flap implant placement,” and “flapless implant placement.”

#### 2.2.1. Searched Databases

A search of health science databases (Cochrane Library and MEDLINE-PubMed) and grey literature was performed, including papers published until May 2013. The most recent electronic search was undertaken on 15 May 2013.

#### 2.2.2. Language

Only articles in English were included in this review.

### 2.3. Data Collection and Analysis

#### 2.3.1. Selection of Studies

The titles and abstracts (when available) of all articles identified through the electronic searches were scanned independently by at least two review authors. For studies appearing to meet the inclusion criteria, or for which there were insufficient data in the title and abstract to make a clear decision, the full report was obtained. The full reports were assessed independently by at least two review authors to establish whether they met the inclusion criteria or not. Disagreements were resolved by discussion. All studies meeting the inclusion criteria then underwent quality assessment and data extraction. Studies rejected at this or subsequent stages were recorded in the “flow diagram of literature review,” and reasons for exclusion were recorded.

#### 2.3.2. Data Extraction

**Figure 1:**
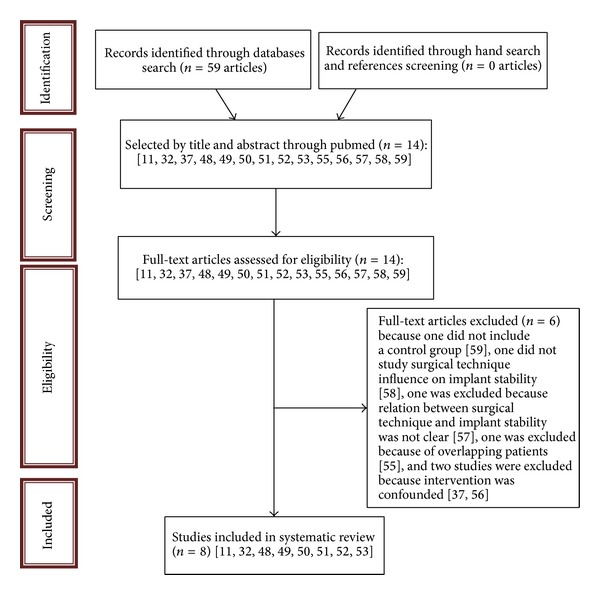
Flow diagram of literature review.

Data were extracted by at least two review authors independently using specially designed data extraction form. Any disagreement was discussed and a third review author was consulted where necessary (Figure 1).

For each study, the following data were extracted (Table 1).

- Year of publishing, country of conducting the study, sample size, and number of implants.
- Implant dimensions, surface treatment, and implant manufacturer.
- Arch region of implant insertion and the surgical technique used.
- Primary stability ISQ or IT values and association between primary stability and surgical technique.
- Confounders included in analysis.
- Secondary stability ISQ or IT values and association between secondary stability and surgical technique.

### 2.4. Assessment of Quality and Control of Bias in Included Studies

The risk of bias assessment for the included studies was considered independently and in duplicate by at least two review authors.

This was conducted using the Methodological checklist for prognostic studies developed by the National Institute for Health and Clinical Excellence of the United Kingdom (2009) [54] (Table 2). Checklist items were worded so that “yes” response always indicates that the study has been designed and conducted in such a way as to minimize the risk of bias for that item. An “unclear” response to a question may arise when the answer to an item is not reported or is not reported clearly. A study was classified as having high methodological quality if at least five of six parameters received the answer “yes,” moderate methodological quality if at least three of the parameters received the answer “yes,” or low methodological quality if two or less parameters received the answer “yes.”

## 3. Results

### 3.1. Description of Studies

See (Table 1).

### 3.2. Characteristics of the Study Settings and Investigators

The search procedure retrieved 59 articles from electronic searches. After screening the titles and abstracts (when available) independently by at least two review authors, 14 articles appeared to meet the inclusion criteria [11, 32, 37, 48–53, 55–59].

Of the 14 potentially eligible studies, 5 studies had to be excluded because relation between surgical technique and implant stability was not clear in one study [57], in two studies intervention was confounded [37, 56], one study did not include a control group [59], and one study did not study the surgical technique influence on implant stability [58].

Thus, a total of 9 clinical studies [11, 32, 48–53, 55] that met the inclusion criteria underwent quality assessment and data extraction.

### 3.3. Characteristics of the Interventions

*(1) Undersized Implant Site Preparation.* Will undersized implant site preparation affect primary and/or secondary stability?

Two observational clinical studies [51, 52] studied this intervention.

A clinical study [51] compared between the undersized implant site preparation and conventional implant site preparation with respect to the primary stability. The implants were inserted in posterior maxilla; six groups with 10 implants each, two controls (C1 and C2), and four tests (T1–T4) were created according to the implant dimensions and the surgical technique adopted. In C1 group, implants of 3.75 mm width and 10 mm length were used with the 2- and 3-mm diameter drills reached up to 10 mm length. In T1 group, implants of 4 mm width and 10 mm length were used, with the 2- and 3-mm diameter drills reached up to 10 mm length. In T2 group, implants of 4 mm width and 10 mm length were used, with the 2-mm dill reached to 10 mm, and the 3-mm drill reached to 7 mm length. In C2 group, implants of 3.75 mm width and 11.5 mm length were used with the 2- and 3-mm diameter drills reached up to 11.5 mm length. In T3 group, implants of 4 mm width and 11.5 mm length were used with the 2- and 3-mm diameter drills reached up to 11.5 mm length. Finally, in T4 group, implants of 4 mm width and 11.5 mm length were used with the 2-mm drill reached up to 11.5 mm and the 3-mm diameter drills reached up to 8.5 mm length. Both ISQ and IT values were recorded at implant placement to evaluate the primary stability.

The other clinical study [52] investigated the effect of undersized drilling technique on primary implant stability when compared with the standard drilling protocol. Undersized bone drilling was performed using 2.8 mm twist drills for 4.1 mm diameter implants; widening of implant bed with osteotome or tapping was not used. Implants with same dimensions (12 × 4.11 mm) were used in both groups. All implants were placed in posterior maxilla and mandible using one stage protocol. ISQ and IT values were recorded at implant placement to evaluate the primary stability.

*(2) Osteotome Technique.* Will osteotome technique affect primary and/or secondary implant stability?

One RCT [49] and two clinical observational studies [48, 50] studied this intervention.

A clinical observational study [48] compared conventional implant placement with osteotome technique to place 10 implants in maxillary anterior region. The implants were placed in the first quadrant using the conventional method (group A) and in the second quadrant using the osteotome technique (group B). For group A the implant sites were sequentially enlarged to 3.7 mm in diameter with pilot and spiral drills according to standard protocol, in group B the implant sites were prepared initially by a 2 mm diameter pilot drill, this was followed by condensing the bone using osteotomes of increasing diameters using a hand ratchet. Once the implant sites were prepared, implants of 13 mm length and 3.7 mm width were inserted. ISQ values of implant stability were measured at implant placement and at six months after.

Another clinical observational study [50] compared osteotome technique with conventional technique for implant placement. A total of 102 implants were placed in posterior segment of maxilla, 51 self-tapping (4 mm in width and 10 mm in length) implants, and 51 non-self-tapping (4.1 mm in width and 10 mm in length). Four study groups were formed according to the surgical technique to be used for implant site preparation and implant macrodesign. In group I: bone condensation technique was used for implant site preparation and self-tapping implants were inserted; group II: non-self-tapping implants were placed following condensation technique; group III: self-tapping implants were inserted after bone drilling; and in group IV: bone drilling was performed and non-self-tapping implants were placed. In groups I and II, bone condensation technique was performed by pilot drill and bone condensers of increasing diameter, while in groups III and IV, implant sites were gradually enlarged with pilot and spiral drills. All implants were placed without pretapping. The study involved one stage surgical protocol. ISQ values were measured at implant placement and after twelfth weeks.

One RCT [49] compared the conventional drilling technique and the osteotome technique in anterior segment of the maxilla. 46 screw type oral implants with the length of 10 or 12 mm and diameter of 4.1 mm were used. For control group, implant bed sites were prepared with pilot and spiral drills to a final diameter of 3.3 mm according to protocol recommended by manufacturer. For the test group after preparing a pilot hole with 2.2 mm diameter drill, the procedure was continued with series of consecutive osteotomes to a final diameter of 3.5 mm according to manufacturer instructions. All implants were placed in the sites using a nonsubmerged technique and in one stage procedure. ISQ values representing implant stability were measured at implant placement and at three months after.

*(3) Piezosurgery.* Will piezosurgery affect primary and/or secondary implant stability?

One RCT [11] compared piezosurgery with conventional implant bed preparation. The trial was conducted using split mouth technique in 20 patients. Each patient received two identical adjacent implants in upper premolar area. The control site was performed with conventional twist drills and the test site was performed with specific piezoelectric inserts. The last instrument used was 3 mm in diameter in both groups to place 4 mm diameter and 10 mm length implants. ISQ values were recorded at implant placement and three months later.

*(4) Flapless Surgical Technique.* Will flapless surgical technique affect primary and/or secondary stability of implants?

One observational clinical study [32] compared placement of implants with flap elevation versus flapless implant placement with respect to primary and secondary implant stability. Forty patients with complete edentulous maxilla were consecutively admitted for treatment with implant supported prosthesis. A computer topography was obtained for the computer-assisted implant planning. One hundred and ten implants were placed conventionally in 23 patients (flap group) and eighty five implants in 17 patients by means of flapless method (flapless group) using a stereolithographic template. The ISQ values were recorded immediately after implant placement and after a period of 3 months.

*(5) Low-Level Laser Irradiation*. Will low-level laser irradiation affect primary and/or secondary stability of implants?

One RCT [53] compared implant placement with low-level laser irradiation with implant conventional placement without laser irradiation. Thirty implants were distributed bilaterally in posterior mandible of eight patients. At the experimental side the implants were submitted to low-level laser treatment and in the control side the irradiation was simulated (placebo). The irradiations were performed with a gallium aluminum-arsenide (GaAlAs) diode low-level laser with continuous emission of 830 nm wavelength. The first irradiation was performed in the immediate post-operation period and was repeated every 48 hours in the first 14 days. ISQ values were recorded initially at implant placement and up to 12 weeks.

### 3.4. Quality Assessment and Control of Bias in Included Studies

None of the articles was excluded from the systematic review after quality assessment, except for one article on studies [50, 55] conducted by the same author and having some overlapping patients. In this case, after ranking the studies, the one with the highest score [50] was included in the systematic review, the other [55] was excluded. A priori calculation for the sample size was undertaken in only two studies [49, 53]. Inclusion and exclusion criteria for the sample selection were clearly defined in only five studies [11, 32, 49, 50, 53]. In addition, blinding of outcome assessors were mentioned by the authors in only three of the included studies [11, 50, 53]. Finally, confounders were not considered for analysis in three studies [32, 49, 52]. Consequently, the quality assessment and control of bias ranked five articles as “moderate” and three as “high” (Table 3).

### 3.5. Effects of Interventions

*(1) Will Undersized Implant Site Preparation Affect Primary and/or Secondary Stability?* In both studies [51, 52], the difference in ISQ and IT values among the undersized drilling and the standard press-fit drilling techniques was not statistically significant (*P* > 0.05), but it was clearly in favour of the undersized group. However, secondary stability was not evaluated.

*(2) Will Osteotome Technique Affect Primary and/or Secondary Stability?* Shayesteh et al. [49] and Marković et al. [50] found positive association between using the osteotome technique and the primary implant stability. They demonstrated a statistically significant higher primary stability for implants placed with osteotome technique than those placed with the conventional drilling technique in the maxillary anterior [49] and maxillary posterior regions [35], based on ISQ values (*P* < 0.05). In contrast, Padmanabhan and Gupta [48], based on ISQ values, demonstrated a statistically significant higher primary stability for implants placed with conventional drilling technique than those placed with osteotome in the maxillary anterior region (*P* < 0.05).

With respect to the influence of osteotome technique on secondary implant stability, there was no significant influence of using osteotome on secondary implant stability when compared with conventional drilling technique for ISQ values, in the two selected studies [48, 49] (*P* > 0.05). On the other hand, Marković et al. [50] showed a statistically significant higher secondary stability for implants placed with osteotome technique than those placed with the conventional drilling technique during the entire 12-week observation period, based on ISQ values (*P* < 0.05).

*(3) Will Piezosugery Affect Primary and/or Secondary Stability?* The single RCT [11] demonstrated that there was no real difference in primary stability when implants were placed following piezoeletric technique versus the conventional twist-drill technique (*P* > 0.05). However, it found a statistically significant higher secondary stability for piezogroup than the control group. This statistically significant difference was during the entire follow-up 90 days, and from day 14 to day 42, in particular, the difference was extremely significant (*P* < 0.0001).

*(4) Will Flapless Surgery Affect Primary and/or Secondary Stability of Implants?* The one observational clinical study [32] demonstrated that there was a positive association between the flapless technique and the primary and secondary implant stability at three months after surgery (*P* < 0.001).

*(5) Will Low-Level Laser Therapy Affect Primary and/or Secondary Stability of Implants?* The one RCT [53] concluded that there was no evidence of any effect of irradiating bone osteotomies with infrared wavelengths on either primary or secondary implant stability within 12-week follow-up in the posterior mandible, when measured by RFA.

## 4. Discussion

The purpose of this systematic review was to evaluate whether there was scientific evidence to support the association between different surgical techniques and primary and/or secondary implant stability. The surgical techniques that we found in the world literature evaluated by clinical studies whether they have influence on primary and/or secondary implant stability were the undersized drilling, the osteotome technique, the piezosurgery, the flapless, and the low-level laser therapy. Just three randomized controlled trials (RCTs) and five observational clinical studies were included. We selected only clinical studies that verified the association between the surgical techniques and implant stability. Laboratory or animal studies which did not report any clinical implant-related outcomes were not considered of interest since they would not be able to provide reliable clinical information for the prognosis of dental implant rehabilitation.

Because only a limited number of studies investigated the influence of different surgical techniques on stability of dental implants, the pattern of the current literature review was customized to primarily summarize the pertinent information.

When evaluating whether the undersized drilling technique could enhance the primary implant stability, the two included observational clinical studies [51, 52] did not show a significant difference between the undersized drilling and the standard press-fit drilling techniques, but it was clearly in favour of the undersized group. The authors concluded that using thinner drills for implant placement in sites with poor bone density (posterior edentulous maxilla and mandible) is beneficial in enhancing primary implant stability. The higher primary stability of implants inserted after undersized drilling compared with those inserted after standard press-fit drilling might be interpreted by that the implants placed in undersized beds could compress the bone and increase its density, thereby enhancing the primary implant stability. However, why no significant difference was detected between the under drilling and the press-fit techniques could be interpreted by the relatively small sample size of those two studies which likely made them underpowered to demonstrate any significant difference in outcome measures between groups. Therefore, further clinical prospective studies and randomized controlled trials with larger sample sizes are required to provide compelling scientific-based evidence of the influence of the undersized drilling technique on the primary and also on the secondary implant stability and healing potential of bone.

When evaluating what the impact of using the osteotome in implant bed preparation on primary and/or secondary implant stability is, only one RCT [49] and two clinical observational studies [48, 50] were selected. Shayesteh et al. [49] and Marković et al. [50] found positive association between using the osteotome technique and the primary implant stability. This increase in primary stability could be due to changes in the micromorphology of peri-implant trabecular bone caused by apicolateral condensation by osteotome. So, the primary stability is enhanced in this low density bone maybe due to increase in its density [4]. In contrast, Padmanabhan and Gupta [48] demonstrated a statistically significant higher primary stability for implants placed with conventional drilling technique than those placed with osteotome in the maxillary anterior region (*P* < 0.05). The too small sample size (*n* = 5) in this study to provide any reliable evidence and methodological differences might be responsible for this contrasting result compared with the abovementioned two.

With respect to the influence of osteotome technique on secondary implant stability, there was no significant influence of using osteotome on secondary implant stability when compared with conventional drilling technique in the two selected studies [48, 49], six months and three months after the surgery, respectively. On the other hand, one article [50] showed a statistically significant higher secondary stability for implants placed with osteotome technique than those placed with the conventional drilling technique during the entire 12-week observation period. Although a direct comparison among the three studies was not possible due to different implant brands used, different recipient sites, and due to different surgeon's experience, an earlier significant increase of secondary stability in the osteotome group in Marković et al. study [50] compared with Padmanabhan and Gupta [48] and Shayesteh et al. [49] studies could be explained by three factors. First: as different surgeon's hands conducted these studies, maybe in Padmanabhan and Gupta [48] and Shayesteh et al. [49] studies, excessive loads were exerted on the bone by osteotome; and provided that loads of more than 20 MPa, which might be anticipated during use of osteotomes, could displace bone marrow spaces and disturb the blood supply, the bone needs more time to form new spaces for angiogenesis [60] and to repair this microdamaged bone [61]. While in Marković et al. study [50], the lateral bone compression might be within the physiological range and as such may have stimulated bone healing probably by activating the trauma-dependent repair mechanism known as “regional acceleratory phenomenon,” unlike the usual process of bone regeneration in the control group. So this might interpret why secondary stability was higher for osteotome technique compared with conventional technique in this study [50]. Second: provided that secondary stability is not only influenced by surgical technique but also by implant surface characteristics [11], the enhanced surface characteristics of implants used in the Marković et al. study [50] might accelerate the bone healing process. Third, the larger sample size in Marković et al. study [50] compared with Padmanabhan and Gupta [48] and Shayesteh et al. [49] might be more able to detect a significant difference between the two techniques.

When evaluating whether using piezosurgery in implant bed preparation could influence the primary and/or secondary implant stability, just one RCT was found [11]. It demonstrated that there was no real difference in primary stability when implants were placed following piezoeletric technique versus the conventional twist-drill technique. However, it found a statistically significant higher secondary stability for piezogroup than the control group. A possible interpretation of the earlier shifting from a decreasing to an increasing stability pattern in ultrasonic preparation sites, when compared with the traditional drilling technique, could derive from the cleaning effect of piezosurgery [13], microvibrations, and the cavitation effect of saline solution could result in effectively removing bony debris and tissue remnants deriving from site preparation, exposing marrow spaces, and favoring a rapid migration of osteoprogenitor cells into the fresh wound [11]. Thus, ultrasounds were effective in stimulating bone healing. However, the results of this study cannot be generalized because of some of limitations. Variables such as the single operator's surgical technique, the limited numerosity of the sample, and the choice of the surgical site (limited to the lateral maxilla) must be taken into account. Also, the implants were not yet loaded and it cannot be stated whether the finding may have a prognostic value for long-term stability of the implants procedure. Therefore, further RCTs using a larger sample size and longer follow-ups are necessary in order to confirm or refute these findings, and, thus, benefit from the possible clinical advantages of piezosurgery in immediate and early loading protocols for dental implant therapy.

When assessing the influence of flapless procedure on primary and/or secondary implant stability, just one observational clinical study was selected [32]. Concluding from this study, there was positive association between the flapless technique and the primary and secondary implant stability at three months after surgery. Interpreting this finding, it can be assumed that raising a mucoperiosteal flap and having the bone denuded during a certain time causes a postsurgical reaction and may have an impact on the bone remodeling around the implant [62]. While the opposite occurs with flapless procedure where the bone remains covered by the periosteum; this may increase vascularity of the peri-implant mucosa, which furthermore appeared to be free from signs of inflammation [63]. Despite that primary and secondary implant stability were observed in slight favor of the flapless method in this study, we cannot generalize this finding because of single operator's surgical technique, the choice of the surgical site (limited to complete edentulous maxilla), and because the implants were not yet loaded and it cannot be stated whether the finding may have a prognostic value for long-term stability of the implants.

When evaluating whether the use of low-level laser therapy (LLLT) to stimulate the osteotomy bone could influence the primary and/or secondary implant stability, only one RCT was conducted [53]. It concluded that there was no evidence of any effect of irradiating bone osteotomies with infrared wavelengths on either primary or secondary implant stability within 12-week follow-up in the posterior mandible. This finding could be explained by a hypothesis that the effect of the laser could have been masked by the high initial stability attained. This high initial stability can be attributed not only to the bone quality (type II bone in the posterior mandible) but also to the implant geometry used in this study. Thus, additional LLLT may have little impact macroscopically. However, it is important to point out that outcomes of this study are limited to the specific methodology and results may differ in different bone conditions and implants when using different LLLT protocols with other methodologies and different lengths of follow-up. In addition, the small sample size of this trial is another limitation.

To provide objective assessment of implant stability, three methods were chosen to assess implant stability in this review: the resonance frequency method, which generated the ISQ value, the percussion method, which generates the PTV value, and the insertion torque measurement that provided the IT value in Ncm. Despite that the Periotest has been the subject of criticism as a result of its poor sensitivity [39] and the insertion torque method allows a single measurement of primary stability and cannot be used for evaluating secondary stability, the three methods were chosen to cover the maximum number of clinical studies on this subject and to avoid subjectivity. Although “Periotest value,” “PTV,” and “Periotest” were used as key words, none of the selected articles used this method to assess primary or secondary stability.

Although this systematic review aimed to verify the influence of different surgical techniques on primary and/or secondary stability of dental implants, it was also possible to extract some data concerning the implant dimensions, implant macrodesign, and the bone density from the selected articles. Turkyilmaz et al. [51] discovered an important influence of implant diameter on primary implant stability (*P* < 0.05). However, Katsoulis et al. [32] showed no significant effect of implant diameter and length on primary and secondary stability. Since implant shape, design, and surface characteristics are important for primary stability [4], most of the selected articles in this review standardized the implant marco- and microdesign except one [50], which demonstrated that self-tapping implants achieved greater primary and secondary stability at 12-week than non-self-tapping implants with conventional bone drilling technique (*P* < 0.05). Also, because there is a positive association between primary implant stability and bone mineral density of the receptor site [7], most of the selected studies in this review utilized one specific surgical site of the arch to minimize the effect of bone density on stability. One of the selected articles [51] reported strong correlations between bone density and primary implant stability values (ISQ and IT). With respect to the influence of gender factor on primary stability, Alghamdi et al. [52] and Katsoulis et al. [32] revealed significantly higher ISQ values for men.

Despite the relative positive association found between primary and/or secondary implant stability and some of the aforementioned surgical techniques, the methodological quality and control of bias of the studies need to be improved to produce stronger evidences. A priori calculation for the sample size was undertaken in only two studies [49, 53]. Inclusion and exclusion criteria for the sample selection were clearly defined in only five studies [11, 32, 49, 50, 53]. In addition, blinding of outcome assessors were mentioned by the authors in only three of the included studies [11, 50, 53]. Finally, confounders were not considered for analysis in three studies [32, 49, 52]. Consequently, the quality assessment and control of bias ranked five articles as “moderate” and just three as “high.”

This systematic review had several limitations. First, the search was limited to English-language publications, which may have introduced a publication bias and excluded other relevant articles. However, such an exclusion may not considerably change the overall estimate of treatment effects [64]. Second, the quality assessment and control of bias ranked five articles as “moderate” as assessed by the Methodological checklist for prognostic studies developed by the National Institute for Health and Clinical Excellence of the United Kingdom [54]. Third, most of the selected articles had small or very small sample sizes, with relatively short follow-ups. Fourth, the inclusion of nonrandomized controlled clinical trials (CCTs) in the analysis may have introduced a bias. However, it was postulated that CCTs can complement the evidence provided by RCTs, particularly when RCTs are not of a high quality [65].

## 5. Conclusions

### 5.1. Implications for Practice

These conclusions are based on few studies with small or very small sample sizes, relatively short follow-ups, moderate methodological quality, and being sometimes judged to be at moderate risk of bias, therefore they should be viewed with great caution.

1. There is a weak evidence suggesting that undersized drilling technique could enhance the primary implant stability in sites of poor bone density.
2. There is still a lack of evidence about the influence of undersized drilling technique on secondary implant stability.
3. There is a weak evidence suggesting that using the osteotome technique to prepare implant beds in poor bone density could enhance the primary and secondary implant stability.
4. There is a weak evidence suggesting that ultrasonic implant site preparation by piezoelectric inserts does not affect the primary mechanical stability but could fasten the bone healing process and increase the secondary implant stability, earlier than the traditional drilling technique.
5. There is a weak evidence suggesting that flapless procedure could enhance the primary and secondary implant stability.
6. There is insufficient evidence supporting or confuting the efficacy of irradiating bone osteotomies with infrared wavelengths for enhancing the primary or secondary stability of the implants.

### 5.2. Implications for Research

More properly designed, RCTs with at least 1-year follow-up after implant loading are needed to understand the influence of undersized drilling, the osteotome technique, the piezosurgery, the flapless, and the low-level laser therapy on primary and secondary stability of implants placed particularly in low density bone. At this time, we could revise the existing loading protocols in this poor-quality bone dealing with these suggested surgical techniques.

## Figures and Tables

**(a):** 

Author and year	Geographical location	Sample	Implant dimensions (mm) and surface	Number of implants	Implant and manufacturer
Padmanabhan and Gupta 2010 [48]	India	Number: 5 Gender: 2 ♀ with a mean age of 29, 3 ♂ with a mean age of 23	Length: 13 Diameter: 3.7 Surface: microgrip 1–5 *μ*m roughness	10	Uniti (Equinox Medical Technologies)

Shayesteh et al. 2013 [49]	Iran	Number: 30 Gender: 18 ♀, 12 ♂ Mean age: 40.5	length: 10, 12 Diameter: 4.1 Surface: sandblasted, large grit, and acid etched	46	SLA oral implants (Straumann AG)

Marković et al. 2013 [50]	Belgrade	Number: 53 Gender: 25 ♀, 28 ♂ Mean age: 43.9	Length: 10 Diameter: 4 mm Surface: BlueSky Bredent-sandbasted and etched osseo connect; Straumann-2–4 *μ*m roughness sandblasted and acid etched	102	51 self-tapping BlueSky (Bredent), 51 non-self-tapping Standard Plus SLActive (Straumann)

Turkyilmaz et al. 2008 [51]	Turkey	Number: 22 Gender: 10 ♀, 12 ♂ Mean age: 49	Lengths: 10, 11.5 Diameters: 3.75, 4 Surface: layer of titanium oxide	60	TiUnite Mk III (Nobel Biocare)

Alghamdi et al. 2011 [52]	Saudi Arabia	Number: 29 Gender: 12 ♀, 17 ♂ Mean age: 47 ± 8	Length: 12 Diameter: 4.1 Surface: 2–4 *μ*m roughness sandblasted and acid etched	52	Standard Plus SLActive (Straumann)

Stacchi et al. 2013 [11]	Italy	Number: 20 Gender: 12 ♂, 8 ♀ Mean age: 59.7 ± 13.6	Length: 10 mm Diameter: 4.0 Surface: nanotite surface	40	NanoTite Parallel Walled Certain (Biomet 3i)

Katsoulis et al. 2012 [32]	Switzerland	Number: 40 Gender: 16 ♀, 24 ♂ Mean age: 61 ± 9	Length: 10, 13 Diameter: 3.5, 4.3 Surface: anodized surface	195	Replace Select Tapered (Nobel Biocare)

García-Morales et al. 2012 [53]	Brazil	Number: 8 Gender: 2 ♂, 6 ♀ Mean age: 36	Diameter: 3.8 Length: 11 Surface: sandblasted and acid etched	30	XiVE-S (Dentsply Friadent)

**(b):** 

Author and year	Regions of implant insertion	Surgical technique	Primary stability: ISQ, PTV and/or IT (N cm) mean (SD)	Confounders included in analysis	Association between Primary stability and surgical technique	Secondary stability: ISQ, PTV and/or IT (N cm) mean (SD)	Association between Secondary stability and surgical technique
Padmanabhan and Gupta 2010 [48]	Maxillary anterior region	Osteotome technique versus conventional drilling	ISQ: drilling 64.77 osteotome 59.60	No confounders cited	ISQ drilling > ISQ osteotome significantly (*P* = 0.026)	ISQ 6 months: drilling 55.40 osteotome 61.50	No significant difference between ISQ drilling and ISQ osteotome 6 months after surgery (*P* = 0.076)

Shayesteh et al. 2013 [49]	Maxillary anterior region	Osteotome technique versus conventional drilling	ISQ: drilling 64.70 osteotome 70.9	Implant length: cited, but not included in analysis	ISQ osteotome > ISQ drilling significantly (*P* = 0.026)	ISQ 3 months: drilling 71.37 osteotome 72.71	No significant difference between ISQ drilling and ISQ osteotome 3 months after surgery (*P* = 0.06)

Marković et al. 2013 [50]	Maxillary posterior region	Osteotome technique versus conventional drilling	ISQ: drilling and non-self-tapping 61.20 ± 1.63 osteotome and non-self-tapping 74.03 ± 3.53 drilling and self-tapping 65.10 ± 3.03 osteotome and self-tapping 74.34 ± 4.09	Implant macrodesign (self-tapping versus non-self-tapping) influenced the stability during the entire follow-up period after bone drilling and only between the 2nd and 12th postoperative weeks, following bone condensation (*P* < 0.05)	ISQ osteotome > ISQ drilling significantly for self-tapping and non-self-tapping implants (*P* < 0.05)	ISQ 12th weeks: drilling and non-self-tapping 67.10 ± 0.32 osteotome and non-self-tapping 71.88 ± 1.10 drilling and self-tapping 68.20 ± 1.81 osteotome and self-tapping 73.54 ± 2.58	ISQ osteotome > ISQ drilling significantly for self-tapping and non-self-tapping implants during the entire 12-week observation period (*P* < 0.05)

Turkyilmaz et al. 2008 [51]	Maxillary posterior region	Undersized drilling versus press-fit drilling	IT for 4 × 10 implants: standard drilling: 35.9 ± 6 undersized drilling 37.2 ± 7 IT for 4 × 11.5 implants: standard drilling: 38.5 ± 7 Undersized drilling 41.1 ± 6	Implant diameter influenced the stability bone density correlated with stability	For 4 × 10 and 4 × 11.5 implants: no significant differences between both (ISQ and IT) standard drilling and both (ISQ and IT) undersized drilling (*P* > 0.05)	Not evaluated	Not evaluated

Alghamdi et al. 2011 [52]	Posterior maxilla and mandible	Undersized drilling versus press-fit drilling	ISQ: standard drilling 66.69 ± 5.41 undersized drilling 68.58 ± 4.81 Maxilla 66.96 ± 5.58 mandible 66.52 ± 5.25 ♀ 64.39 ± 5.15 ♂ 68.27 ± 4.85 IT: standard drilling 34.62 ± 5.82 undersized drilling 35.19 ± 4.79 maxilla 34.07 ± 4.81 mandible 34.20 ± 4.93 ♀ 33.48 (±4.63) ♂ 36.38 (±5.96)	Bone density and jaw position (maxilla versus mandible): cited but not included in analysis and were not accounted for to remove their confounding influence on surgical techniques between groups	No significant differences between both (ISQ and IT) standard drilling and (ISQ and IT) undersized drilling (*P* > 0.05) ISQ ♂ > ISQ ♀ significantly (*P* < 0.001) No significant difference for IT values between women and men (*P* > 0.05)	Not evaluated	Not evaluated

Stacchi et al. 2013 [11]	Maxillary premolar area	Piezosurgery versus conventional drilling	ISQ: drills 72.2 ± 5.8 piezoelectric 70.5 ± 5.8	No confounders cited	No significant difference between ISQ drills and ISQ piezoelectric (*P* = 0.3215)	ISQ 3 months: drills 69.2 ± 5.5 piezoelectric 71.0 ± 2.9	ISQ piezoelectric > ISQ drills significantly during the entire period of observation (90 days): from day 14 to day 42, in particular, the difference was extremely significant (*P* < 0.0001)

Katsoulis et al. 2012 [32]	Complete edentulous maxilla	Flapless versus flap procedure	ISQ: flap 57.7 (±1.8) flapless 62.1 (±1.8) ♀ 56.5 (±2.0) ♂ 61.4 (±1.4) 10 mm length: 60.5 (±3.0) 13 mm length: 58.7 (±1.3) 3.5 mm diameter: 58.0 (±2.0) 4.3 mm diameter: 59.1 (±1.5)	Implant diameter and length did not influence stability Bone density not evaluated	ISQ standard > ISQ flapless significantly (*P* < 0.001) ISQ ♂ > ISQ ♀ significantly (*P* = 0.01)	ISQ 3 months: Flap 56.0 (±2.0) flapless 65.4 (±1.7) ♀ 55.9 (2.4) ♂ 62.0 (2.0) 10 mm length: 59.5 (4.1) 13 mm length: 59.6 (1.9) 3.5 mm diameter: 60.2 (2.7) 4.3 mm diameter: 59.0 (2.2)	ISQ flap > ISQ flapless significantly at 3 months (*P* < 0.001) ISQ ♂ > ISQ ♀ significantly at 3 months (*P* < 0.001)

García-Morales et al. 2012 [53]	Mandibular posterior region	Low-level laser stimulation versus placebo	ISQ: conventional 75.7 (5.6) laser 77.4 (3.4)	No confounders cited	No significant difference between ISQ conventional and ISQ laser (*P* < 0.05)	ISQ 12 weeks: conventional 78.4 (3.0) laser 76.3 (4.1)	No significant difference between ISQ conventional and ISQ laser at 12 weeks (*P* > 0.05)

**Table 2:** Methodological checklist for prognostic studies developed by the National Institute for Health and Clinical Excellence from United Kingdom [54].

Study identification				
Circle one option for each question				
(1.1)	The study sample represents the population of interest with regard to key characteristics, sufficient to limit potential bias to the results. To minimize bias, the study population should be clearly defined and described and should represent the source population of interest. Points to consider include the following. Is the source population or the population of interest adequately described with respect to key characteristics? Are the sampling frame and recruitment adequately described, possibly including methods to identify the sample (number and type used; e.g., referral patterns in healthcare), period of recruitment and place of recruitment (setting and geographical location)? Are inclusion and exclusion criteria adequately described (e.g., including explicit diagnostic criteria or a description of participants at the start of the follow-up period)? Is participation in the study by eligible individuals adequate? Is the baseline study sample (i.e., individuals entering the study) adequately described with respect to key characteristics?	Yes	No	Unclear

(1.2)	Loss of follow-up is unrelated to key characteristics (i.e., the study data adequately represent the sample), sufficient to limit potential bias. To minimize bias, completeness of follow-up should be described and adequate. Points to consider include the following. Is the response rate (i.e., proportion of study sample completing the study and providing outcome data) adequate? Are attempts to collect information on participants who dropped out of the study described? Are reasons for loss to follow-up provided? Are the key characteristics of participants lost to follow-up adequately described? Are there any important differences in key characteristics and outcomes between participants who completed the study and those who did not?	Yes	no	unclear

(1.3)	The prognostic factor of interest is adequately measured in study participants, sufficient to limit potential bias. To minimize bias, prognostic factors should have been defined and measured appropriately. Points to consider include the following. Is a clear definition or description of the prognostic factor(s) measured provided (including dose, level, duration of exposure, and clear specification of the measurement)? Are continuous variables reported, or appropriate cut-off points (i.e., not data-dependent) used? Are the prognostic factor measured and the method of measurement valid and reliable enough to limit misclassification bias? (This may include relevant outside sources of information on measurement properties, as well as characteristics such as blind measurement and limited reliance on recall). Are complete data for prognostic factors available for an adequate proportion of the study sample? Are the method and setting of measurement the same for all study participants? Are appropriate methods employed if amputation is used for missing data on prognostic factors?	Yes	No	Unclear

(1.4)	The outcome of interest is adequately measured in study participants, sufficient to limit bias. Is a clear definition of the outcome of interest provided, including duration of follow-up? Are the outcome that was measured and the method of measurement valid and reliable enough to limit misclassification bias? (This may include relevant outside sources of information on measurement properties, as well as characteristics such as “blind” measurement and limited reliance on recall.) Are the method and setting of measurement the same for all study participants?	Yes	No	Unclear

(1.5)	Important potential confounders are appropriately accounted for, limiting potential bias with respect to the prognostic factor of interest. To minimize bias, important confounders, should be defined and measured, and confounding should be accounted for in the design or analysis. Points to consider include the following. Are all important confounders, including treatments (key variables in the conceptual model), measured? Are clear definitions of the important confounders measured (including dose, level and duration of exposures) provided? Is measurement of all important confounders valid and reliable? (This may include relevant outside sources of information on measurement properties, as well as characteristics such as “blind” measurement and limited reliance on recall.) Are the method and setting of measurement of confounders the same for all study participants? Are appropriate methods employed if imputation is used for missing data on confounders? Are important potential confounders accounted for in the study design (e.g., matching for key variables, stratification or initial assembly of complete groups)? Are important potential confounders accounted for in the analysis (i.e., appropriate adjustment)?	Yes	No	Unclear

(1.6)	The statistical analysis is appropriate for the design of the study, limiting potential for the presentation of invalid results. To minimize bias, the statistical analysis undertaken should be clearly described and appropriate for the design of the study. Points to consider include the following. Is the presentation of data sufficient to assess the adequacy of the analysis? Where several prognostic factors are investigated? Is the strategy for model building (i.e., the inclusion of variables) appropriate and based on a conceptual framework or model? Is the selected model adequate for the design of the study? Is there any selective reporting of results? Are only prespecified hypotheses investigated in the analyses?	Yes	No	Unclear

It was used to perform the quality assessment and control of bias				

**Table 3:** 

	Padmanabhan and Gupta 2010 [48]	Shayesteh et al. 2013 [49]	Marković et al. 2011 [55]	Stacchi et al. 2013 [11]	Turkyilmaz et al. 2008 [51]	Marković et al. 2013 [50]	Alghamdi et al. 2011 [52]	Katsoulis et al. 2012 [32]	García-Morales et al. 2012 [53]
(1) The study sample represents the population of interest with regard to key characteristics, sufficient to limit potential bias to the results	Unclear	Yes	Unclear	Unclear	Unclear	Unclear	Unclear	Unclear	Yes

(2) Loss to follow-up is unrelated to key characteristics (i.e., the study data adequately represent the sample), sufficient to limit potential bias	Yes	Unclear	Yes	Yes	Yes	Yes	Yes	Yes	Yes

(3) The prognostic factor of interest is adequately measured in study participants, sufficient to limit potential bias. (n these studies the prognostic factor was the surgical technique)	Yes	Yes	Yes	Yes	Yes	Unclear	Yes	Yes	Yes

(4) The outcome of interest is adequately measured in study participants, sufficient to limit bias. (The outcome was the primary and/or secondary stability)	Unclear	Unclear	Yes	Yes	Unclear	Yes	Unclear	Unclear	Yes

(5) Important potential confounders are appropriately accounted for, limiting potential bias with respect to the prognostic factor of interest. (e.g., implant dimensions and bone density)	Yes	No	Yes	Yes	Yes	Yes	No	No	Yes

(6) The statistical analysis is appropriate for the design of the study, limiting potential for the presentation of invalid results	Yes	Yes	Yes	Yes	Yes	Yes	Yes	yes	Yes

Category and situation of the article	4 ‘‘yes:” moderate methodol quality included	3 ‘‘yes:” Moderate methodol quality included	5 ‘‘yes:” High methodol quality excluded∗	5 “yes:” High methodol quality included	4 ‘‘yes:” Moderate methodol quality included	4 ‘‘yes:” Moderate methodol quality included	3 ‘‘yes:” Moderate methodol quality included	3 ‘‘yes:” Moderate methodol quality included	6 “yes:” high methodological quality included

*The articles conducted by the same author had some overlapping patients. After ranking these studies, the one with the highest score was included in the systematic review, the others were excluded.
